# Inhibitory control of the excitatory/inhibitory balance in psychiatric disorders

**DOI:** 10.12688/f1000research.12155.1

**Published:** 2018-01-08

**Authors:** Martijn Selten, Hans van Bokhoven, Nael Nadif Kasri

**Affiliations:** 1 Centre for Developmental Neurobiology, Institute of Psychiatry, Psychology and Neuroscience, New Hunt's House, Guy's Campus, King’s College London, London, SE1 1UL, UK; 2 MRC Centre for Neurodevelopmental Disorders, New Hunt's House, Guy's Campus, King’s College London, London, SE1 1UL, UK; 3 Department of Human Genetics & Department of Cognitive Neuroscience, Radboudumc, Geert Grooteplein 10, Box 9101, 6500 HB Nijmegen, Netherlands; 4 Donders Institute for Brain, Cognition, and Behaviour, Centre for Neuroscience, 6525 AJ Nijmegen, Netherlands

**Keywords:** psychiatric disorders, PV basket cells, PV interneurons, chandelier cells

## Abstract

Neuronal networks consist of different types of neurons that all play their own role in order to maintain proper network function. The two main types of neurons segregate in excitatory and inhibitory neurons, which together regulate the flow of information through the network. It has been proposed that changes in the relative strength in these two opposing forces underlie the symptoms observed in psychiatric disorders, including autism and schizophrenia. Here, we review the role of alterations to the function of the inhibitory system as a cause of psychiatric disorders. First, we explore both patient and post-mortem evidence of inhibitory deficiency. We then discuss the function of different interneuron subtypes in the network and focus on the central role of a specific class of inhibitory neurons, parvalbumin-positive interneurons. Finally, we discuss genes known to be affected in different disorders and the effects that mutations in these genes have on the inhibitory system in cortex and hippocampus. We conclude that alterations to the inhibitory system are consistently identified in animal models of psychiatric disorders and, more specifically, that mutations affecting the function of parvalbumin-positive interneurons seem to play a central role in the symptoms observed in these disorders.

## Introduction

Psychiatric disorders, including autism, schizophrenia, bipolar disorder, attention deficit hyperactivity disorder (ADHD) and depression, affect millions of people and are a major socio-economic burden ^1– 3^. The identification of underlying genetic defects and risk factors is becoming increasingly efficient because of genome-wide interrogation methodologies, yet owing to the complex multifactorial origin of most cases, a conclusive molecular diagnosis is made for only a minority of patients. Therefore, the underlying causes for these conditions are poorly understood, and often treatment is still based on symptomology ^4– 6^. In 2003, Rubenstein and Merzenich proposed autism spectrum disorders (ASDs) to be caused by an increase in the ratio between excitation and inhibition, called the E/I balance ^7^. Since then, this hypothesis has been substantiated by a vast number of studies and also has been implicated in other psychiatric disorders such as schizophrenia ^8^, consistent with their partially overlapping phenotypes ^9^. Recently, the focus has shifted to changes to the inhibitory side of the E/I balance ^10, 11^, in particular to one class of inhibitory neurons, parvalbumin (PV)-positive interneurons ^12^. In this review, we focus on the role of the inhibitory system in psychiatric disorders and explore the changes to the inhibitory systems in different disorders. We then discuss the role and function of PV interneurons and highlight the changes to this specific class of interneurons in the various psychiatric disorders.

## Evidence for inhibitory dysfunction in psychiatric disorders

Since Rubenstein and Merzenich postulated their hypothesis of a reduced E/I balance in ASDs, there has been an increasing amount of evidence for disrupted inhibitory control in psychiatric disorders. This evidence comes from post-mortem studies and studies of patient phenotypes.

Firstly, post-mortem studies on patient brains have revealed consistent changes to the inhibitory system in various disorders. Studies of autistic brains revealed reduced expression of the gamma-aminobutyric acid (GABA) synthesizing enzymes GAD65 and GAD67, as well as various GABA receptor subunits, in parietal cortex and cerebellum ^13, 14^. In schizophrenia, reductions of interneuron markers have been found in the prefrontal cortex ^15– 18^, a region strongly implicated in this condition ^19^. Interestingly, in recent years, this reduction has been shown to be caused by a reduction of the expression of the interneuron markers rather than a reduction of the number of interneurons ^20– 22^, which indicates reduced activity of these neurons ^23– 25^. In addition, both increased and decreased numbers of specific interneuronal subtypes are reported in bipolar disorder ^17, 26^, while a reduced inhibitory function is reported in depression ^27, 28^ and bipolar disorder ^29^.

Secondly, patients with psychiatric disorders display phenotypes that are strongly correlated to impaired inhibition. Epilepsy is a common comorbidity with psychiatric disorders and has consistently been linked to impaired inhibitory function ^30– 33^. In patients with autism, it is estimated that the prevalence of epilepsy comorbidity is around 25% ^34, 35^. However, this is dependent on the type of autism, and the prevalence can be as high as 80% in Rett syndrome ^36^, a monogenic form of autism caused by mutation in the *MeCP2* gene ^37^. It is currently unclear whether schizophrenia is a risk factor for epilepsy. A limited number of studies have been dedicated to this question, and contradicting results have been reported ^38, 39^. However, patients with epilepsy show an increased risk of schizophrenia or schizophrenia-like psychosis ^40^. Likewise, patients with epilepsy show an increased risk for ADHD ^41, 42^.

Another recurrent phenotypic change is the altered power of gamma oscillations, as measured with electroencephalography or magnetoencephalography in humans, indicating changes in neuronal synchrony ^43^. Gamma oscillations are important for integration of information in neuronal circuits and have been linked to various functions, including attention ^44^ and memory ^45^. It was shown that PV-positive interneurons ^46^, specifically PV-positive basket cells (see below), play an important role in these osciliations ^43, 47, 48^. Changes in gamma oscillations are consistently found in patients with schizophrenia ^49^, affecting different regions, including the prefrontal cortex ^50, 51^. Interestingly, while a decrease in gamma power is linked to negative symptoms of this disorder, such as psychomotor poverty ^52^, increased gamma power has been observed during positive symptoms, such as hallucinations ^53^. In addition, computational studies suggest a central role for inhibitory synaptic scaling in maintaining a stable neuronal network ^54^ and found changes in inhibitory transmission to be sufficient to explain the changes in gamma oscillations in schizophrenia ^55^. Together, altered inhibitory control is believed to lead to a change in the power of gamma oscillations, which play a central role in schizophrenia ^56^.

Though studied mainly in schizophrenia, changes in gamma oscillations have been observed in other psychiatric disorders, including autism, ADHD and bipolar disorder ^57– 63^. For example, children with autism show a reduced gamma frequency modulation to a visual task ^64^, whereas in ADHD, increased power and synchrony were observed ^59– 62^. Together, post-mortem and patient studies point to an important role for altered inhibitory function in various psychiatric disorders and indicate a vital role for inhibition in the maintenance of the E/I balance in the healthy brain.

## The central role of parvalbumin-positive interneurons in E/I balance

Cortical and hippocampal synaptic inhibition is mediated by inhibitory interneurons, most of which use GABA as their neurotransmitter. While interneurons make up around 20% of the total neuronal population, they are highly diverse ^65, 66^. For example, different classes of interneurons are specialized to target the dendrites, soma or axon initial segment (AIS) of pyramidal neurons ^65^. This large variety of cell types is believed to illustrate the distinct functions that these cells have in regulation of the network ^67^. Cortical interneurons can be segregated in three non-overlapping groups by means of specific markers: PV, somatostatin (SOM) and the serotonin receptor 3a (5HT3aR), accounting for 40%, 30% and 30% of the total interneuron population, respectively ^68^. 5HT3aR-positive cells mainly originate from the caudal ganglionic eminence and are further divided as vasoactive intestinal peptide (VIP)-positive and VIP-negative interneurons ^68^. VIP-positive interneurons mainly inhibit other interneurons and play an important role in disinhibition of the local circuit ^69^, where they receive excitatory input from other cortical areas ^70, 71^. VIP cells mainly inhibit SOM cells ^72^ but also target PV interneurons ^23^ and are involved in the regulation of the behavioural state of the network ^71, 73^. Recent studies have suggested a direct inhibition by VIP interneurons of pyramidal cells in cortex ^74, 75^. Despite the prominent, mainly disinhibitory, function of VIP cells in the network, only a limited number of studies have implicated VIP cells to be involved in psychiatric disorders ^26, 76^.

SOM interneurons are a diverse class of interneurons originating from the medial ganglionic eminence (MGE) ^68^. These interneurons target non-SOM interneurons ^72^ as well as the dendritic domain of pyramidal neurons, including dendritic spines ^77^. SOM interneurons regulate the integration of local excitatory input ^78, 79^ and have been shown to regulate synaptic plasticity via the control of dendritic calcium spikes in pyramidal cells, affecting learning tasks ^80^. Increasing evidence implicates SOM interneurons in psychiatric disorders. Disinhibition of SOM interneurons leads to an anti-depressive–like phenotype in mice ^81^, and reduced levels of SOM in cerebral spinal fluid have been linked to major depression and mood disorders ^82^. In addition, a recent article shows a role for SOM interneurons in gamma oscillations in the visual cortex ^83^, hinting towards a possible role for SOM interneurons in the changes in gamma oscillations observed in psychiatric disorders.

PV interneurons are MGE-derived and are electrophysiologically identified by their fast-spiking phenotype. Although PV interneurons make up only a small part of the entire neuronal population ^84, 85^, these interneurons are strongly implicated in psychiatric disorders and have been shown to play an important role in the regulations of the E/I balance ^8, 10, 86^. PV interneurons are involved in gamma oscillations (see above), and various mutations in disease-linked genes affect PV interneuron function (discussed below) ( Table 1). Different subtypes of PV interneurons are distinguished: basket cells, chandelier cells, bistratisfied cells, and, in hippocampus, oriens-alveus-lacunosum-moleculare cells ^87, 88^ form the largest of these groups, the first two of which are most widely studied. Here, we will discuss both types and focus on their respective roles in the network.

**Table 1.** Genes linked to psychiatric disorders affect distinct subcellular aspects.

Aspect	Gene	Syndrome/Disorder	Model	Investigated region	Phenotype	Reference
Input	*Erbb4*	SZ	PV interneuron KO	Hippocampus	Reduced excitatory input to PV basket cells and chandelier cells	120
	*Nrg1*	SZ	NRG1 treatment of dissociated cortical cultures	Cortical (cultures)	Increased excitatory synapse number onto interneurons	121
	*Fmr1*	Fragile X syndrome; ASD	*Fmr1* KO mouse	Cortex	Reduced local excitatory input onto FS interneurons	122
	*DISC1*	SZ, ASD, depressive disorder, BD	PV-specific shRNA KD *in vivo*	Cortex	Increased excitatory input onto PV interneurons	123
	*Nlgn3*	ASD	PV interneuron KO	Hippocampus Hippocampus	Decreased NMDAR responses Increased glutamate release onto PV interneurons	124
	*Mecp2*	Rett syndrome; ASD	PV interneuron KO	Cortex	Reduced local excitatory input onto PV interneurons	125
Intrinsic	*Mecp2*	Rett syndrome; ASD	PV interneuron KO	Cortex	Increased intrinsic excitability of PV interneurons	125
	*Dysbindin*	SZ	*Dysbindin* KO mouse	Cortex	Reduced excitability of FS interneurons	126
	*Scn1a*	Dravet syndrome; ASD	*Scn1a* KO mouse	Hippocampus	Impaired action potential kinetics in interneurons	127
	*Shank3*	ASD, SZ	*Shank3B* KO mouse	Cortex, Striatum	Reduced activity of PV interneurons	22
Output	*Erbb4*	SZ	PV interneuron KO	Hippocampus	Reduced cartridges from chandelier cells onto pyramidal neurons	120
	*Nrg1*	SZ	Overexpression in pyramidal neurons	Cortex	Increased basket cell and chandelier cell boutons onto pyramidal neurons	128
	*Tsc1*	Tuberous sclerosis; ASD	Sparse *Tsc1* deletion in CA1 pyramidal neurons	Hippocampus	Reduced inhibitory synaptic strength onto pyramidal neurons	129
	*Ube3a*	Angelman syndrome; ASD	Maternal loss of *Ube3a* mouse	Cortex	Reduced inhibitory drive from FS and non-FS interneurons onto pyramidal neurons	130
	*Shank3*	ASD, SZ	*Shank3-exon9* KO mice	Cortex	Reduced inhibitory input onto pyramidal neurons	131
			*Shank3-exon9* KO mice	Hippocampus	Increased inhibitory input onto pyramidal neurons	131
	*Git1*	ADHD	*Git1* KO mouse	Hippocampus	Reduced inhibitory inputs onto pyramidal neurons	132
	*Cdh13*	ADHD	*Cdh13* KO mouse	Hippocampus	Increased number of inhibitory synapses onto pyramidal neurons	133
	*Nlgn 2*	ASD	*Nlgn2* KO mouse	Cortex	Reduced inhibitory drive onto pyramidal neurons from FS interneurons	134
		ASD	*Nlgn2* KO mouse	Hippocampus	Reduced number of perisomatic synapses onto pyramidal neurons	135
	*Nlgn 3*	ASD	*Nlgn3* R451C mouse	Hippocampus	Reduced inhibitory drive from PV basket cells onto pyramidal neurons	136
	*Cntnap2*	ASD	*shRNA* KD in dissociated cortical cultures	Cortical (cultures)	Reduced inhibitory drive onto pyramidal neurons	137

ADHD, attention deficit hyperactivity disorder; ASD, autism spectrum disorder; BD, bipolar disorder; FS, fast-spiking; KD, knockdown; KO, knockout; PV, parvalbumin-positive; shRNA, short hairpin RNA; SZ, schizophrenia.

### PV basket cells

Basket cells are the largest group of PV interneurons and specifically target the soma and proximal dendrite of pyramidal neurons ^87^. The perisomatic location of these axon terminals allows PV basket cells to have a strong control over the excitability of pyramidal neurons. Among other cortical inputs, PV basket cells receive the same excitatory input as their pyramidal cell targets, wiring the basket cell into a feed-forward circuit: excitatory input will excite both the PV basket cell and the pyramidal neuron, followed by the PV basket cell inhibiting the pyramidal neuron. The delay between the excitatory and inhibitory input onto the pyramidal cell creates a coincidence detection window, in which excitatory input can summate to elicit an action potential in the pyramidal cell ^89^. If inhibitory input arrives at the pyramidal cell before an action potential is evoked, the somatic targeted GABA action will prevent action potential initiation. So PV basket cells allow action potential initiation in pyramidal neurons only if the excitatory information is time-locked and of sufficient strength.

In order to mediate fast inhibition, PV basket cells are optimized for fast signalling ^85^. Action potentials are initiated at the AIS and propagate at high velocities through the axon ^90^ which is enriched for the fast sodium channel Na _V_1.1 ^91^. Synaptically, calcium inflow is mediated by fast P/Q-type calcium channels ^92^, which are located directly adjacent to the release site ^93^. The post-synaptic site, on the pyramidal neuron, contains the fast GABAAα1 receptor subunit ^94^. These fast properties ensure an optimal speed of PV basket cell signalling and tightly regulate coincidence detection windows of pyramidal neurons.

### Chandelier cells

Chandelier cells, or axo-axonic cells, are a group of interneurons that target the AIS of pyramidal neurons ^95, 96^. These cells form vertically oriented clusters of axon terminals, called cartridges, giving them a chandelier-like appearance. A single pyramidal cell receives contacts from multiple chandelier cells ^97^, forming an average of 3 to 5 boutons each ^98^ depending on the brain region ^99^ and age ^100^. The synapses are enriched for the GABAAα2 receptor subunit ^101, 102^ and pre-synaptically express the high-affinity GABA transporter 1 (GAT1) ^103^. The function of chandelier cells in the network remains largely unknown. However, chandelier cell activity has been shown to increase with increasing network activity ^104^. In addition, the axon terminals of chandelier cells are specifically absent from the epileptic focus ^105, 106^, and pharmacological induction of seizures leads to a loss of chandelier cells in rats ^107^, suggesting a role in preventing excessive excitatory activity in the network for these interneurons ^104^.

Since their discovery, there has been a debate about the actions of chandelier cells (reviewed by Wang and colleagues ^98^). Some studies using brain slice recordings showed a depolarizing ^108^ and even excitatory ^109^ action for these interneurons, whereas others report an inhibitory action ^110^. However, chandelier cell membrane potential fluctuations resembling *in vivo* patterns appear strongly inhibitory ^111^, and recordings *in vivo* also suggest an inhibitory role for these interneurons ^112, 113^.

**Figure 1.**
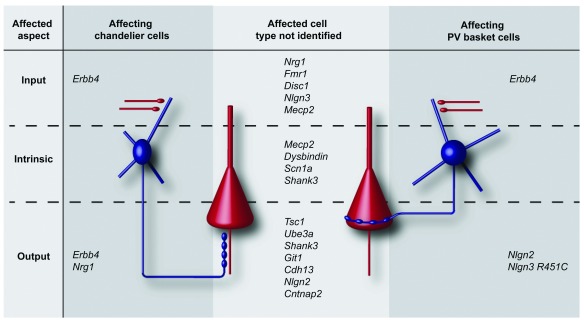
Genes linked to psychiatric disorders affect inhibition on different subcellular aspects. (Left) Genes affecting the input, intrinsic properties or output of chandelier cells. (Right) Genes affecting the input, intrinsic properties or output of parvalbumin-positive (PV) basket cells. (Middle) Genes affecting input, intrinsic properties or output of interneurons, without the interneuron subtype being identified. Pyramidal cells are shown in red, and interneurons are shown in blue.

Whereas patient studies of schizophrenia patients consistently identify both a reduction in the number or length of chandelier cell cartridges ^114, 115^ as well as the misregulation of proteins associated with chandelier cell synapses ^12, 116– 118^, mouse research on this interneuron class is hampered by the absence of a strategy to specifically target these interneurons. As a result, a limited number of studies focus on chandelier cells but instead report on PV interneurons in general, or PV basket cells, which provide a more accessible target of study because of their relative abundance ( Figure 1). Nonetheless, chandelier cells are considered to play an important role in psychiatric disorders ^11, 98, 119^, and the development of strategies to specifically target this interneuron subtype would be an important step towards understanding the role of these interneurons.

Targeting the perisomatic region (basket cells) or AIS (chandelier cells) gives these interneurons strong control over pyramidal cell excitability, and regulation of the synaptic strength of these interneurons is important for normal function of the network. For example, Xue and colleagues have shown that pyramidal neurons receive an amount of synaptic inhibition that is proportional to the amount of synaptic excitation they receive ^138^, maintaining the E/I balance on the pyramidal cell. Manipulation of pyramidal cell activity leads to a compensatory change in inhibitory drive onto these cells, specifically from PV interneurons ^138^. In addition, PV interneuron activity is reduced during learning and increased during fear conditioning, and an experimental increase of PV interneuron activity leads to impaired learning ^23^. Apart from synaptic connections, basket cells ^139^ and chandelier cells ^111, 140^ connect via electrical connections, called gap junctions. This electrical coupling synchronizes the interneurons ^139^, which in turn allows them to synchronize the network ^141^ (for example, in gamma oscillations ^142^).

Together, these studies indicate that control of the E/I balance by PV interneurons is important for normal network function and that PV interneuron-mediated inhibition can be regulated upon alterations in the network state. Changes in PV interneuron-mediated inhibition would shift the E/I balance and lead to a disruption in network function. Indeed, various parameters affecting inhibitory function of PV cells are consistently found to be altered in psychiatric disorders. In the next section, we focus on studies on animal models of these conditions and discuss how different changes affecting PV cell activity lead to a shift of the E/I balance ( Table 1).

## Altered PV interneuron activity is caused by changes to different subcellular aspects

Alterations to the inhibitory drive, affecting the E/I balance, can arise in different ways. Reduced excitatory input onto interneurons, reduced intrinsic excitability of interneurons, and a reduction in inhibitory synapse number or strength onto pyramidal cells all result in a shift of the E/I balance towards excitation. Indeed, patient and animal studies of psychiatric disorders consistently report changes affecting inhibitory function. A complicating factor in the interpretation of these results comes from the dynamic ability of neuronal networks to adapt to changes, known as homeostatic plasticity. Homeostatic plasticity is the ability of neurons to maintain their levels of excitability within a narrow range and is a constantly active feedback process ^143^. For example, classic experiments have shown that blocking of action potentials leads to a strengthening of excitatory ^144^ and a weakening of inhibitory synapses ^145^. Apart from their synaptic input, neurons can regulate their intrinsic excitability ^146^, which is observed both in excitatory and inhibitory neurons in culture ^147^ and *in vivo* ^148^. This means that genetic mutations affecting a specific neuronal property in a specific cell type can trigger homeostatic processes affecting other properties or cell types. In this way, mutations affecting both inhibitory or excitatory cells could ultimately affect inhibitory function ^86^. It is therefore difficult to distinguish the direct effect of a gene related to a psychiatric disorder from the network adaptation it causes.

Nonetheless, changes to the function of PV interneurons, either direct or indirect, are consistently observed in psychiatric disorders affecting input, output and intrinsic properties, which all lead to an altered inhibitory action of these cells onto their targets ( Figure 1).

### Changes to the input onto PV interneurons

Excitatory inputs onto neurons drive their excitability. Depending on the brain region, PV interneurons receive various types of excitatory input ^85^, and the amount of excitatory inputs onto PV interneurons is dynamically regulated by behaviour ^23^. Changes in the amount of excitation onto PV interneurons, altering their activity, have been reported in mouse models of various psychiatric disorders.

The tyrosine kinase receptor ErbB4 has been identified as a risk gene for schizophrenia in genome-wide association studies (GWASs) ^149, 150^. In the adult mouse, expression of ErbB4 is restricted to interneurons ^128, 151^ and localizes to both the axon terminals ^128, 152^ and post-synaptic densities ^128, 151^. Selective removal of ErbB4 from PV interneurons causes a reduction in excitatory synapses formed onto both PV basket cells and chandelier cells as well as a reduced number of PV synapses formed on pyramidal neurons ^120, 121^. This reduced input and output connectivity of PV interneurons is indicative of a reduced inhibitory drive onto pyramidal neurons. As a result of this reduced inhibition of the pyramidal neurons, these neurons become more active, as was seen from the increased frequency of excitatory inputs to both pyramidal neurons and PV interneurons ^120^. Consequently, recording of the local field potential *in vivo* revealed a hyperactive network and increased gamma oscillations ^120^. Single-nucleotide polymorphisms in neuregulin 1 (NRG1), a ligand of ErbB4, have been implicated in schizophrenia ^153^ and bipolar disorder ^154, 155^. Treatment of neuronal cultures with NRG1, activating ErbB4, leads to an increase in excitatory synapses formed onto interneurons ^121^. Together, these data show that ErbB4 signalling plays an important role in the regulation of excitatory synapse number onto PV interneurons and that disruption of this system leads to a shift of the E/I balance towards excitation ^120^.

Also, studies on animal models of ASD have reported postsynaptic changes on PV interneurons. Fragile X syndrome is caused by reduced or absent levels of the RNA-binding protein FMRP, leading to intellectual disability and, in about half of the affected males, ASD ^156, 157^. While changes to long-term depression on excitatory cells have been the centre of attention for this condition ^158^, changes to the inhibitory system have consistently been identified ^159, 160^. *Fmr1* knockout (KO) mice show a reduced expression of GABAA receptor subunits ^161^ as well as a reduction in the number of PV interneurons ^162^. In addition, these mice show a marked reduction in local, but not thalamic, excitatory input onto fast-spiking interneurons in layer 4 of the somatosensory cortex, while both the connectivity of fast-spiking interneurons onto pyramidal neurons and excitatory inputs onto pyramidal neurons were unaltered ^122^. Consistently, the resulting reduced inhibitory drive from fast-spiking interneurons was accompanied by reduced synchrony of gamma oscillations ^122^. These changes point towards an altered E/I balance towards excitation in fragile X syndrome ^163^.

Mutations in another gene linked to autism, the transcriptional modulator methyl-CpG-binding protein 2 ( *MECP2*), the causative gene for Rett syndrome ^37^, show a similar phenotype. Selective removal of *Mecp2* from PV cells leads to a specific reduction of local excitatory input, but not thalamocortical input, onto these cells in layer 4 of the visual cortex at post-natal day (P) 30 ^125^. In addition, experimentally evoked PV interneuron input onto pyramidal cells was unaltered ^125^. Calcium imaging revealed that these synaptic changes lead to a reduced visually evoked, but not spontaneous, activity of PV interneurons ^125^. Paired recordings at P15 revealed an increased inhibition from PV interneurons through an earlier maturation in *Mecp2* KO mice ^164^, and this earlier maturation might influence network development through interference with the normal critical period ^164, 165^, leading to the changes observed at later ages. This notion is consistent with the recent idea that developmental changes might play an important role in the development of psychiatric symptoms later in life ^166^. The exact contribution of PV interneurons to the phenotypes observed in Rett syndrome is still unclear. While a general interneuron removal of *Mecp2* does recapitulate most Rett syndrome phenotypes ^167^, studies removing *Mecp2* specifically from PV interneurons have been able to replicate only some of these phenotypes ^168^ or none at all ^125^. Of note, selective removal of *Mecp2* from SOM interneurons also recapitulates part of the Rett syndrome phenotypes ^168^, and selective removal of *Mecp2* from either PV interneurons or SOM interneurons has been reported to cause circuit-wide deficits in information processing ^169^. From these studies, a picture is emerging in which excitatory inputs onto PV interneurons are found to be altered in different psychiatric disorders, leading to a reduced activity of these neurons and thereby tilting the E/I balance towards excitation.

### Changes to the intrinsic properties of PV interneurons

The intrinsic properties of neurons are important in translating input into output. Altering these properties allows the neurons to regulate their excitability ^170, 171^, through which they play an important role in the maintenance of the E/I balance ^143^. Whereas some of these changes might be causative to psychiatric disorders, others are believed to be compensatory. For example, selective deletion of *Mecp2* from PV interneurons leads to an increased membrane potential, as well as a hyperpolarized action potential threshold in these cells at P30 ^125^, but only a slight hyperpolarization of the membrane potential at P15 ^164^. These changes increase the cell’s excitability and are most likely compensatory for the reduced excitatory synaptic input described above ^125^ but could still act towards deficits in information processing observed in these animals ^169^.

Family-based association data have identified dysbindin ( *DTNBP1*) as a susceptibility gene for schizophrenia ^172^, whose dominant circuit impact is impaired inhibition ^173^. Dysbindin KO mice show a reduced number of PV interneurons in hippocampal CA1 ^173, 174^, and transcriptome changes of various proteins involved the regulation of intrinsic properties ^174^. Recordings from PV interneurons in dysbindin KO mice show a reduction in action potential frequency resulting in a reduced inhibitory drive ^126^. Interestingly, dopamine D2-receptor expression is increased in these mice, and application of the D2-receptor antagonist quinpirole increases PV interneuron action potential frequency more in dysbindin KO mice than in wild-type mice, suggesting that the changes in action potential frequency are compensatory ^126^.

Intrinsic changes can also be the primary cause of psychiatric disorders. Single-gene mutations in *SCN1A*, encoding the sodium channel Na _V_1.1α subunit, give rise to Dravet syndrome, a rare genetic epileptic encephalopathy. Patients with Dravet syndrome suffer from epilepsy and have an increased risk for autism ^175, 176^. Na _V_1.1 is enriched in the AIS of inhibitory neurons ^177^, primarily of PV interneurons ^91^, where axon potentials are initiated ^178^. Interneurons from *Scn1a* heterozygous and KO mice show reduced firing frequencies to current injections as well as a reduced action potential amplitude and an increased action potential width, indicating a reduced inhibitory control over downstream targets ^127^. Removal of *Scn1a* specifically from forebrain interneurons ^179^ or PV interneurons specifically ^180^ recapitulates phenotypes found in patients. In addition, increasing GABA signalling by application of the positive allosteric GABA _A_ receptor modulator clonazepam was sufficient to rescue the abnormal social behaviour in *Scn1a* ^+/−^ mice ^181^. These data show that loss of SCN1A primarily affects interneurons and that the consequently reduced inhibitory drive plays an important role in Dravet syndrome.

Recently, a new hypothesis has been proposed in the field of schizophrenia, focussing on the myelination of PV interneurons as a point of pathological convergence ^182^. As discussed above, PV cells play a central role in schizophrenia. Myelination abnormalities, including white matter abnormalities ^183^, reduced numbers of oligodendrocytes ^184^ and post-mortem gene expression analysis ^185^, have been identified in schizophrenia. Myelination of PV interneurons has been observed in rats ^186^, mice ^187^ and post-mortem in humans ^188^. Myelination is important for fast action potential propagation ^189^, and deficits in the myelination of PV interneurons are proposed to disrupt inhibitory network function ^182^. However, this appealing hypothesis remains to be experimentally tested.

## Changes to synapses formed by PV interneurons

Changes to PV synapses are abundantly studied and identified in various conditions. Post-mortem studies of schizophrenia patients consistently identify changes to the cartridges formed by chandelier cells, showing a decrease in pre-synaptic GAT1 expression ^116, 117^ and an increase in the expression of post-synaptic GABAAα2 ^118^. These changes would lead to an increased inhibitory drive from chandelier cells and are believed to be compensatory for a reduced activity of these cells, as indicated by reduced levels of GAD67 in PV cells ^190^. In addition, a recent study shows a reduction in the density of a specific type a cartridges, calbindin-positive cartridges, in schizophrenia ^114^.

Besides changes in the input to PV interneurons, mutations in *Erbb4* also lead to a reduction in synapses formed by PV interneurons on pyramidal neurons, specifically from chandelier cells ^120, 128^. In addition, overexpression of *Nrg1*, the ligand for ErbB4, in pyramidal neurons increases bouton density on both the AIS and the soma of pyramidal neurons. The increase in bouton density on the AIS originates from chandelier cells since only these cells target the AIS. The origin of the increase in perisomatic bouton density is not clear since a recent report has shown that synapses formed by cholecystokinin (CCK) basket cells require ErbB4 function to form perisomatic synapses ^191^. Future research should unveil whether the increase in perisomatic boutons density arises from PV or CCK basket cells.

Tuberous sclerosis is a disorder whose symptoms include epilepsy and autism ^192^. Loss-of-function mutations in the mammalian target of rapamycin (mTOR)-negative regulators TSC1 or TSC2 underlie this condition ^193^. Slice recordings from *TSC1* KO neurons revealed a reduced inhibitory drive onto CA1 pyramidal neurons, while excitatory input was unaltered ^129^. *TSC1* KO neurons were created by sparsely targeting neurons in a conditional TSC1 KO mouse with a cre-expressing adeno-associated virus ^129^. The finding that sparse KO of TSC1 leads to a similar phenotype as in neurons from full KO animals indicates that these results are cell-autonomous rather than compensatory ^129^.

Angelman syndrome, caused by loss-of-function mutations or deletion of E3 ubiquitin ligase *UBE3A* ^194^, is characterized by epilepsy and autism ^195, 196^. Mouse models for Angelman syndrome recapitulate human phenotypes ^197^ and initially were found to show a reduced excitatory drive onto pyramidal neurons ^198^. However, inhibitory input was also found to be decreased, caused by defects in synaptic vesicle cycling ^130^. It was hypothesized that the reduced inhibition could outweigh the reduced excitation, leading to the epilepsy and autism phenotypes obeserved ^130, 199^. Consistent with this idea, a recent study using *in vivo* whole cell recordings shows that pyramidal neurons in *Ube3a* KO mice display decreased orientation selectivity ^200^, indicative of reduced inhibition ^201, 202^. However, these mice also show increased excitability of pyramidal neurons, which is non–cell-autonomous, suggesting that pyramidal neurons homeostatically increase their excitability because of a relative decrease of excitation ^200^. While it is unclear whether reduced inhibition or reduced excitation has a stronger impact on pyramidal neurons in Angelman syndrome, the change in their relative contribution, resulting in an altered E/I balance, seems to play a pivotal role in this condition.

Single *SHANK3* mutations, at the level of point mutations or microdeletions, have been identified in patients with ASD ^203^. In the human genome, there are three SHANK genes ( *SHANK1-3*), which all code for scaffold proteins located at the postsynaptic density of excitatory synapses, of which SHANK3 is best studied ^204– 206^. Because of this localisation, most studies have focussed on excitatory synapses, where *Shank3*-deficient mice show reduced cortico-striatal connectivity ^207^, impaired long-term potentiation ^208^ and reduced GluA1 expression ^209^. Recent studies, however, indicate that the inhibitory system is also affected. *Shank3* mutant mice lacking exon 9 show reduced inhibitory input onto layer 2/3 pyramidal neurons but an increase of these events in CA1 pyramidal neurons ^131^. In addition, PV levels are reduced in *Shank1* KO and *Shank3b* KO mice, indicating reduced activity ^22^.

Presynaptic neurexins and their postsynaptic partners neuroligins are a large class of cell-adhesion molecules that have been shown to play important roles in synaptic specificity ^210^. Overexpression or knockdown of neuroligins leads to an increase or decrease in synapse number, respectively ^211^. Neuroligins are expressed at specific synapses: neuroligin ** 1 (NLGN1) is mainly expressed at excitatory synapses ^212^, NLGN2 is expressed at inhibitory synapses ^213^, NLGN3 ** is expressed at both inhibitory and excitatory synapses ^214^ and NLGN4 ** is expressed at glycinergic synapses ^215^. Mutations and deletions affecting human *NLGN3*, including a gain-of-function mutation, and *NLGN4 de novo* mutations have been found in Swedish families and have been associated with autism ^216^. *Nlgn3* deletion in mice leads to increased inhibitory transmission onto pyramidal neurons ^217^, specifically from CCK-positive interneurons because of an increased tonic endocannabinoid signalling, whereas PV interneuron connectivity is unaffected ^136^. A recent study, however, has shown that conditional deletion of *Ngln3* from PV interneurons alters AMPA/NMDA (alpha-amino-3-hydroxy-5-methyl-4-isoxazolepropionic acid/N-methyl-d-aspartate) ratio of excitatory input onto these cells and causes reduced gamma oscillations ^124^. In addition to the loss of *NLGN3*, a gain-of-function amino acid substitution (R451C) in *NLGN3* is associated with autism ^216^. Mice carrying this mutation show a strong reduction in inhibitory drive from PV interneurons while increasing the inhibitory drive from CCK cells ^136^.

*Nlgn4* KO mice show a reduced number of perisomatic inhibitory synapses in hippocampus and a concomitant reduction in inhibitory input ^218^. In addition, a reduced power of evoked gamma oscillations in acute slices was observed ^218^. Different mutations in *NLGN2* have been linked to autism ^219^ and schizophrenia ^220^. Deletion of *Nlgn2* has been shown to specifically reduce the amount of perisomatic synapses on pyramidal neurons in hippocampus ^135^ and reduce inhibitory transmission from PV interneurons but not SOM interneurons ^134^.

Neurexins are less well studied in the context of psychiatric disorders. There are three neurexin genes, each coding for an α- and β-neurexin. Mutations in neurexin ( *NRXN1*) have been associated with autism ^221^ and schizophrenia, affecting both *NRXN1α* and *NRXN1β* ^222, 223^. *Nrxn1α* KO mice do not show changes in inhibitory drive but do show a reduced excitatory drive onto CA1 pyramidal neurons ^224^. However, mice carrying mutations in *Nrxn1β* show a reduced frequency of both inhibitory and excitatory input onto cortical pyramidal neurons ^225^ suggestive of a reduced number of synaptic contacts. These studies show that neuroligin and neurexin mutations that are linked to autism affect the inhibitory system, including perisomatic-targeting interneurons.

Studies of ADHD rodent models have mainly focussed on the dopamine system and excitatory synapses ^226^. However, recent studies identify changes to inhibitory connectivity. G protein–coupled receptor kinase interacting protein 1 (GIT1) has been identified by a GWAS as a risk gene for ADHD ^132^, but recent studies challenge this claim ^227, 228^. *Git1* KO mice show ADHD-like phenotypes, including hyperactivity ^132^. While excitatory input to CA1 pyramidal neurons remains unaltered, the frequency of inhibitory inputs is reduced, suggesting a reduction of inhibitory synaptic contacts ^132^, consistent with previous studies showing a role for GIT1 in inhibitory synapses ^229^. In addition, PV expression was reduced while other interneuron markers remained unaltered ^132^.

Another ADHD-linked gene identified by GWASs, cadherin 13 ( *CDH13*) ^230– 232^, is exclusively expressed in inhibitory neurons ^133^. *Cdh13* KO mice show an increase in inhibitory synaptic contacts onto CA1 pyramidal neurons, while excitatory inputs remained unaltered ^133^. This increase in inhibitory synaptic contacts could underlie the increase in gamma oscillations observed in ADHD patients discussed before ^59– 62^. *Cdh13* KO mice show a reduced number of interneurons at embryonic day 18.5 ^233^ but not at P21 ^133^. Experiments are needed to test whether the changes in interneuron number have a role in the aetiology of ADHD.

In addition to changes in the number and strength of inhibitory synapses, changes are found in the modulation of inhibitory synaptic transmission. Metabotropic GABA receptors, GABA _B_ receptors, are expressed both pre- and postsynaptically in GABAergic synapses ^234^. Postsynaptic GABA _B_ receptors activate potassium channels that hyperpolarize the postsynaptic cell ^235^. Presynaptic GABA _B_ receptors, in addition, can reduce calcium inflow and reduce neurotransmitter release ^236, 237^. A reduction of GABA _B_ subunit expression has been observed in post-mortem studies of patients with schizophrenia ^238, 239^, patients with bipolar disorder ^240^ and patients with major depression ^241^ as well as animal models for schizophrenia ^242– 244^. However, more research is required to identify the exact role for GABA _B_ receptors in psychiatric disorders.

In summary, changes to the inhibitory drive of PV interneurons can be caused by changes affecting the input, output or intrinsic properties of PV interneurons, and animal models of various psychiatric disorders all show alterations to one or more of these aspects, tilting the E/I balance.

## Conclusions

Psychiatric disorders are a diverse group of disorders, but changes to the inhibitory system seem to be a point of convergence. Impairment of normal inhibitory function can arise from input to, output from, or intrinsic properties of inhibitory neurons. Altered inhibitory activity or drive leads to changes in signal processing, which in turn is believed to underlie the phenotypic changes observed in the various psychiatric disorders. PV-positive interneurons play a pivotal role in these conditions, possibly through their strong inhibitory effect on pyramidal cell activity due to the axonal or perisomatic targeting of their axons in combination with the nature of their functions in the network.

Changes to either excitation or inhibition will change the ratio between these two types of input, leading to a change in the E/I balance. The examples described above indicate that various psychiatric disorders occur following changes to the input, output or intrinsic properties of specific interneurons, PV interneurons, leading to an altered activity of these neurons. It seems from the studies discussed that specific changes in the E/I balance lead to a disruption of specific function(s) in the network that affect signal processing in a specific way to result in a specific psychiatric phenotype. This might explain why different disorders present different phenotypes, in both patients and animal studies. For example, changes in chandelier cell cartridges seem to be more prominent in schizophrenia. However, other factors are likely to contribute to the development of a specific disorder. Apart from the affected PV cell type, the direction of the altered activity (increased/decreased E/I balance) might be an important factor. Another interesting aspect might be the changes in interneuron-interneuron connectivity, which could lead to altered signal integration and network activity. Some genes have been associated with multiple psychiatric disorders, indicating that a mutation does not in all cases lead to a specific condition. Individual difference in compensatory plasticity could subtly affect network development, steering the developing network towards a specific disorder. It should be noted that while homeostatic changes might compensate a specific alteration, this compensation might disrupt other pathways. While the above-mentioned factors potentially all play a role in the development of specific psychiatric disorders, more research is needed to identify how specific alterations to PV interneurons affect network processing and behaviour.

The notion that psychiatric disorders are caused by changes to the inhibitory drive from PV interneurons means that a restoration of this drive could improve patient symptoms. An interesting possibility of counteracting the changes to the inhibitory system would be to make use of the neuron’s ability for homeostatic plasticity ^170^. Homeostatic mechanisms function to keep neurons in an optimal range of activity ^245^. Understanding which genetic and molecular mechanisms underlie these homeostatic processes opens the possibility to ‘hijack’ these pathways and manipulate the neuron’s activity in a way to compensate for the altered inhibitory drive. In this way, it might be possible to selectively up- or downregulate the activity levels of specific interneuron populations and thereby ameliorate the symptoms observed in patients with the various disorders.

While psychiatric disorders are still far from being fully understood, the study of the role of the inhibitory system in these conditions might further increase our understanding of both the diseased and the healthy brain and hopefully could lead to treatment or alleviation of the symptoms for those suffering from these conditions.
